# A Potential Role of the Spike Protein in Neurodegenerative Diseases: A Narrative Review

**DOI:** 10.7759/cureus.34872

**Published:** 2023-02-11

**Authors:** Stephanie Seneff, Anthony M Kyriakopoulos, Greg Nigh, Peter A McCullough

**Affiliations:** 1 Computer Science and Artificial Intelligence Laboratory, Massachusetts Institute of Technology, Cambridge, USA; 2 Reasearch and Development, Nasco AD Biotechnology Laboratory, Piraeus, GRC; 3 Naturopathy, Immersion Health, Portland, USA; 4 Internal Medicine, Truth for Health Foundation, Tucson, USA

**Keywords:** neurodegeneration, amyloidosis, g quadruplexes, diabetes, cd16+ monocytes, exosomes, mrna vaccines, prion disease, spike protein, sars-cov-2

## Abstract

Human prion protein and prion-like protein misfolding are widely recognized as playing a causal role in many neurodegenerative diseases. Based on in vitro and in vivo experimental evidence relating to prion and prion-like disease, we extrapolate from the compelling evidence that the spike glycoprotein of SARS-CoV-2 contains extended amino acid sequences characteristic of a prion-like protein to infer its potential to cause neurodegenerative disease. We propose that vaccine-induced spike protein synthesis can facilitate the accumulation of toxic prion-like fibrils in neurons. We outline various pathways through which these proteins could be expected to distribute throughout the body. We review both cellular pathologies and the expression of disease that could become more frequent in those who have undergone mRNA vaccination. Specifically, we describe the spike protein’s contributions, via its prion-like properties, to neuroinflammation and neurodegenerative diseases; to clotting disorders within the vasculature; to further disease risk due to suppressed prion protein regulation in the context of widely prevalent insulin resistance; and to other health complications. We explain why these prion-like characteristics are more relevant to vaccine-related mRNA-induced spike proteins than natural infection with SARS-CoV-2. We note with an optimism an apparent loss of prion-like properties among the current Omicron variants. We acknowledge that the chain of pathological events described throughout this paper is only hypothetical and not yet verified. We also acknowledge that the evidence we usher in, while grounded in the research literature, is currently largely circumstantial, not direct. Finally, we describe the implications of our findings for the general public, and we briefly discuss public health recommendations we feel need urgent consideration.

An earlier version of this article was previously posted to the Authorea preprint server on August 16, 2022.

## Introduction and background

Prion diseases, also known as transmissible spongiform encephalopathies (TSEs), are a group of rare, consistently fatal brain diseases affecting animals and humans. They are caused by 'proteinaceous infectious particles,' which can facilitate disease spread in the absence of a classical infection by a living organism. They include the familiar mad cow disease (bovine spongiform encephalopathy), scrapie in sheep, and chronic wasting disease (CWD) in deer. The primary human prion disease is known as Creutzfeldt-Jakob disease (CJD), and it is always fatal. Fatal familial insomnia (FFI) is a rare fatal genetic disease caused by certain mutations in the prion protein. In common nomenclature, the naturally folded form of the prion protein is referred to as PrP^C^, whereas the misfolded form is called PrP^SC^ (for "scrapie"). Disease propagation occurs through an autocatalytic process whereby external misfolded prion proteins (PrP^SC^) act as an infectious agent to facilitate misfolding of the same protein expressed in neurons. It is now generally recognized that an intermediate soluble oligomeric form of the protein is the toxic agent. In contrast, the insoluble plaque may even be protective in that it results in the clearing of the soluble oligomers [1]. It is increasingly becoming apparent that there is a generalization of prion diseases that can encompass neurodegenerative diseases such as Alzheimer's, Parkinson's disease, and amyotrophic lateral sclerosis (ALS), which are also associated with misfolded proteins that accumulate in plaques and Lewy bodies. These proteins, which are termed amyloidogenic, have also been labeled as "prion-like," and their spread may also have properties that overlap with the classic stricter definition of the prion protein (PrP) [2,3]. Both Tau and α-synuclein have been characterized as amyloidogenic [4-6]. Furthermore, researchers are finding that the TAR-DNA binding protein of 43 kDa (TDP-43), a protein that misfolds in association with ALS, forms aggregates that propagate between cells in a prion-like manner [7]. Protein aggregates can be transmitted from one cell to another through at least three distinct mechanisms: tunneling nanotubes, secretion as naked aggregates, or packaging up into extracellular vesicles such as exosomes [8].

A remarkable study involved a mutated variant of a bacterial plasmid initiator protein, RepA, that builds intracellular amyloid oligomers triggering a lethal cascade in bacteria, similar to the mitochondrial impairment of human cells in neurodegeneration [9]. In this study, the authors worked with a human neuroblastoma cell line engineered to express wild-type RepA. A variant of RepA with an A31V mutation was shown to be prone to forming amyloid fibrils. The genetically modified human neuroblastoma cells were cocultured with murine cells releasing mutated RepA aggregates. The amyloid fibrils released from the murine cells were shown to be present in the cytosol of human recipient cells and were cytotoxic. Thus, the amyloidosis was propagated from the murine cells to the human cells. Based on their results, these authors stated a "central principle of underlying prion biology." "No matter the biological origin of a given prion-like protein, it can be transmitted to a phylogenetically unrelated recipient cell, provided that the latter expresses a soluble protein onto which the incoming protein can readily template its amyloid conformation" [9]. They stated that the intercellular exchange of prion-like protein aggregates could be a common phenomenon. The above paper sets the stage for the narrative we propose here, namely, that the spike protein produced in excess by human cells transfected with the mRNA vaccines, analogous to the mutated prion-like bacterial protein, could be released as amyloid fibrils and taken up by neurons expressing various amyloidogenic proteins, most notably the prion protein, acting as a seed. This could result in cytotoxicity and neurodegeneration, explaining many of the neurological symptoms observed as adverse reactions to the mRNA vaccines. In the remainder of this paper, we further develop this hypothesis based on extensive evidence from the research literature. The idea that the spike protein might be neurotoxic through prion-like capabilities is not new. In 2020, Tavassoly O et al. proposed that peptides derived from the spike protein might cross-seed with existing amyloidogenic human proteins to accelerate fibril formation and cause neurodegeneration [10]. Strong evidence for the prion-like potential of the spike protein comes from a paper published in 2021 that demonstrated through computational modeling that the receptor binding domain (RBD) of the S1 segment of the spike protein, binds to heparin and several different amyloidogenic heparin-binding proteins, including amyloid β, α-synuclein, Tau, the prion protein and TAR DNA-binding protein 43 (TDP-43) [11]. They proposed that this could be the mechanism by which the spike protein could cause mitochondrial oxidative stress, apoptosis, and neurodegeneration. Heparin-binding accelerates the aggregation of amyloid proteins in the brain [12]. A case has been made for amplifying central nervous system damage in Alzheimer's disease due to the endocytosis of spike protein by endothelial cells in the brain in association with COVID-19 infection. Consistent with other studies, these authors proposed that spike-induced endothelialitis disturbs the blood-brain barrier and exacerbates Alzheimer's disease via the interaction of the spike protein with amyloid β or hyperphosphorylated tau [13].

## Review

The spike protein has prion-like domains

The COVID-19 mRNA vaccines are based on lipid nanoparticles containing mRNA encoding the SARS-CoV-2 spike glycoprotein. The vaccine has been engineered in several ways to protect the mRNA contents from breakdown and to assure that cells transfected with it produce large quantities of spike protein at a high production rate over a long time period [14-18]. A comprehensive study using bioinformatics has identified a large number of viral proteins from diverse species that have prion-like signatures in their genetic sequence. In particular, they identified prion-like domains in viral surface proteins involved in receptor binding and fusion with the host cell [19]. These same authors later published a paper analyzing the spike protein's prion-like potential. They found a prion-like domain in the RBD of the SARS-COV-2 spike protein, which was missing from the original SARS-CoV virus. Asparagine (N) and glutamine (Q)-rich regions are characteristic features of many prion proteins. Five amino-acid substitutions in the SARS-CoV-2 variant compared with SARS-CoV formed a hydrophobic Q/N-rich region enabling prionogenesis. They also analyzed some of the SARS-CoV-2 variants, determining that the Delta variant had a higher score for prionogenesis than the original Wuhan strain, whereas Omicron had a substantially lower score [20]. Glutamine-asparagine-rich regions (QNRs) have been found frequently in regulatory molecules and RNA-binding proteins. They are associated with proteins linked to neurodegenerative diseases, including Alzheimer's, Huntington's, and ALS [21]. Hidden Markov Modeling (HMM) is a statistical tool used to compute the likelihood of a particular observation sequence, given a set of training data representing the class under investigation. One of its many applications is evaluating compositional similarities among proteins and prion sequences. An online application, prion-like amino-acid composition (PLAAC) analysis (http://plaac.wi.mit.edu/) employs HMM technology in order to identify candidate prionogenic domains in proteins of viruses, prokaryotes, and eukaryotes [22,23]. The resulting log-likelihood ratio (LLR) obtained using PLAAC is an estimate of the likelihood that an investigated protein is prionogenic. The PLAAC algorithm analyses the prionogenic domains (PrDs) of a given protein that compositionally matches at least one domain of the already investigated yeast prions [24]. Table 1 includes the main eukaryotic (human) pathogenic virus orders and species that contain the most PrD domains estimated by PLAAC and mirrored by their mean LLR scores [19,20,25-28]. In addition, the main SARS coronavirus and its variant LLR scores are also included in Table 1.

**Table 1:** Comparison of prionogenic domains (PrD) amongst eukaryotic DNA, various human pathogenic RNA viruses, and several variants of human pathogenic RNA coronaviruses. LLR: Log-likelihood ratio predicting enrichment of prionogenic domains (PrD) in viral proteins. * : Source [14]; ** : Source [20]; *** : Source [15]. 4* : Source [21]; Source [22]; 5* : Source [23].

	Eukaryotic Human DNA and RNA viruses*	Pathogenic SARS Coronaviruses & Variants***			
Main virus orders and species presented with the highest numbers of prionogenic domains in their proteins	Herpevirales, including α, β & γ Herpesviruses: Mean LLR: 6.75; Megavirales^**: ^Mean LLR: 10.35; Positive strand RNA viruses, including: Picornavirales (Enterovirus B, cardiovirus B) and Flaviviridae (Zika virus, Hepatitis C virus): Mean LLR: 6.47; Baculoviridae (including coronaviruses): Mean LLR: comparable to Herpesviruses.	SARS-CoV: LLR: 4.426	SARS-CoV-2: LLR: 4.856	Delta B.1.617.1 Variant: LLR: 6.025	Omicron B.1.1529 Variant: LLR: 3.080
Protein Domains	For DNA viruses: Viral membrane and envelope involved in glycoproteins and membrane protein. For RNA viruses: Viral precursor proteins and proteins for cell entry	Spike Protein Heptad Repeat 1 (HR-1)	Spike Protein Receptor Binding Domain (RBD)	Spike Protein Receptor Binding Domain	Spike Protein Receptor Binding Domain
Function of proteins	Transcription, translation and protein synthesis. Viral adsorption and entry	Cell adhesion, fusion, cell entry and pathogenesis of SARS CoV infection^4*^	Binding to the ACE2 receptor, adhesion, cell entry and pathogenesis of COVID-19^5*^		

A study evaluating the amyloidogenic potential of the spike protein used both theoretical and experimental methods to verify that the SARS-CoV-2 spike protein can cause amyloid-like fibrils to appear after the protein has been subjected to proteolysis. Theoretical predictions identified seven potentially amyloidogenic sequences within the spike protein. In laboratory experiments where the protein was incubated with the protease neutrophil elastase, amyloid-like fibrils appeared during 24 hours of coincubation. A specific segment, spike 194-213 (FKNIDGYFKI), was highly abundant after six hours, and it overlapped almost completely with the most amyloidogenic sequence identified theoretically. Neutrophils responding to immune activation release neutrophil elastase into the medium, where it would have access to the spike protein and be able to break it down into the amyloidogenic segments [29]. The SARS-CoV-2 spike protein has a unique polybasic four-amino-acid insert, RRAR, at the junction of the S1 and S2 segments, which is not present in SARS-CoV. This sequence has several cleavage sites susceptible to proteases such as neutrophil elastase [30]. Neutrophil elastases secreted by neutrophils attracted to the sites of inflammation caused by the SARS-CoV-2 spike protein can presumably cleave the encountered spike protein and release the S1 segment into the circulation, potentiating an amylogenic response. Neutrophil elastase-derived amyloidosis has long been implicated in amyloidogenesis. Amyloid fibrils trigger the release of neutrophil extracellular traps (NETs) containing elastase from neutrophils, which can induce amyloid fragmentation into toxic oligomers [31]. α-Synuclein is a well-established causative factor in Parkinson's disease (PD). Lewy body dementia (LBD) is a relatively common disease associated with α-synuclein deposits in the brain, showing in many cases similarities with PD [32]. Experimental relationships emerging from bio-informatics studies show that the SARS-CoV-2 spike protein interacts with amyloidogenic proteins, particularly α-synuclein, and it induces Lewy-body-like pathology in a cell line [33]. It also induced upregulation of α-synuclein expression. The authors suggested that these properties of the spike protein could be the underlying mechanism accounting for the link between COVID-19 and PD, which has been found in other studies [34]. Overexpression of α-synuclein increases its risk of misfolding into toxic amyloid fibrils [35]. A case study of a man in his sixth decade of life documented the onset of CJD symptoms coinciding with SARS-CoV-2 infection. The rapid progression of neurological symptoms led to his death two months later [36]. The authors hypothesized that a severe inflammatory response to SARS-CoV-2 either precipitates or exacerbates neurodegenerative disease. A review paper documented this case and several other cases from the published research literature on neurological diseases or syndromes in association with COVID-19 [37]. Their Table 1 lists 25 cases of Alzheimer's disease, epilepsy, multiple sclerosis, prion disease, and visual disturbances associated with long COVID.

Prof. Luc Montagnier is a recently deceased Nobel-prize winner for his work on HIV. A recently published paper co-authored by Montagnier describes 26 cases where the patient became severely ill with spontaneous symptoms of CJD shortly after a COVID-19 vaccine. Twenty-three out of the 26 cases developed symptoms within 15 days of their second injection of an mRNA vaccine. The other three cases were associated with the AstraZeneca DNA vector vaccine, and symptoms appeared within the first month. Of the 26, 20 had died at the time of writing the paper, and the remaining six were in critical condition. The mean time to death was under five months after the injection [38]. CJD is an extremely rare disease, usually affecting only one in a million people in their lifetime. It also usually takes several years from the first onset of symptoms until death. So, this is clearly an extraordinarily unusual type of CJD that should raise a concern about the safety of these vaccines. While still a hypothesis that remains to be proved by human clinical studies, it is possible that this rapidly progressing form of CJD is due to molecular mimicry between the spike protein and the prion protein. Proteins containing prion-like domains are recently being extensively investigated by computational analyses for their causative role not only for prion-like disease development but also for cancer and viral infections [39]. There is a five-residue sequence in the spike protein, YQAGS, that differs by only one residue from a sequence in the C-terminal domain of the prion protein (YQRGS). The sequence in the spike protein is at the end of a B-cell epitope within the RBD. Antibodies to this sequence might bind the C-terminal of the prion protein due to molecular mimicry. Studies have shown that autoantibodies in the globular C-terminal domain can cause an aggressive form of CJD by interfering with the transport of the prion protein into the endoplasmic reticulum [40].

Biodistribution of mRNA vaccines

As early as 1979, it was recognized that exposure of mice to scrapie prion protein, regardless of whether it was via intraperitoneal, IV, or multiple subcutaneous routes, always showed the same pattern of spread of infectivity. Propagation in the spleen consistently showed up well before a noticeable spread to the spinal cord, with infectivity in the brain requiring the longest incubation period. A conclusion was that immune cells carry the infectious proteins into the spleen, and further spread of the infectivity occurs mainly along nerves rather than via the vasculature or the lymphatic system [41].

Unlike PrP, which is highly expressed in the nervous system but expressed at much lower levels in a plethora of other tissues, the amyloid precursor protein (APP) is highly expressed in many tissues apart from the nervous system, including muscles, the liver, the immune system (thymus and spleen) and many other organs [42,43].

Only a few studies have been conducted to examine the biodistribution of mRNA from vaccines subsequent to injection. A study published in 2017 tracked the distribution of mRNA coding for influenza haemagglutinin proteins following injection into mouse muscle. They quantified the maximum level of mRNA found in various organs and used these data to infer the migration pathway of the mRNA. As expected, by far, the highest concentration remained in the muscle (5,680 ng/mL). However, a substantial amount was found in the proximal lymph nodes (2,120 ng/mL), with significantly smaller amounts in the distal lymph nodes (177.0 ng/mL). Among the organs, the spleen and liver had by far the highest concentrations (86.9 ng/mL in the spleen and 47.2 ng/mL in the liver). Smaller amounts were found in the plasma (5.47 ng/mL), bone marrow (3.35 ng/mL), ileum (3.54 ng/mL), and testes 2.37 ng/mL, with trace amounts in many other organs, including the brain (0.429 ng/mL) [44].

Another study tracked the biodistribution pathway of a rabies mRNA vaccine administered intramuscularly to rats. They found that the mRNA appeared in the draining lymph nodes within one day and was also found in blood, lungs, spleen, and liver [45]. Developers of the technology are pleased to see that the mRNA shows up in the lymph system and the spleen because T-cell activation and antibody production by B cells mainly occurs in germinal centers in the lymph nodes and spleen [46].

We recognize and acknowledge that these are animal-based biodistribution studies. As such, their relevance to humans is speculative. These studies are conducted, though, on the expectation that they have some relevance to human biodistribution. In the absence of human studies that would clarify this topic, we include the in vivo studies as the best available evidence that is potentially analogous to human biodistribution.

Exosomes and microRNAs

Exosomes are membranous secreted nanovesicles 30-150 nm in size, generated and released by all cells, often under conditions of stress. These extracellular vesicles are produced in late endosomes by the inward budding of the endosomal membrane. Their cargo is diverse and can include nucleic acids, proteins, lipids, and metabolites. They mediate both near and long-distance intercellular communication through contents that include signaling molecules, nutrients, and toxins. In particular, their lipid membrane can protect internalized RNA molecules from degradation by extracellular ribonucleases. A paper published by Wei H et al. in 2021 provides an excellent review of the complex mechanisms that control sorting proteins, RNAs, and other molecules into exosomes for export and delivery to other cells [47]. It has been shown experimentally that cells that take up mRNA from the nanoparticles in mRNA vaccines package up some of the mRNAs, together with the ionizable cationic lipids, into small lipid particles that are then released into the external medium as exosomes. In fact, these authors found a 1:1 ratio of cationic lipid molecules to nucleotides in the released exosomes [48]. They also demonstrated that cells that took up the exosomes could synthesize protein from the mRNA contained in the exosomes. This experiment involved mRNA that codes for human erythropoietin, but a similar result can be expected for the spike-encoding mRNA of the COVID-19 vaccines. In theory, this means that an immune cell in the spleen could ship intact mRNA coding for the spike protein up to the brain along the vagus nerve, and a neuron or microglial cell in the brain could take up the mRNA and begin synthesizing spike protein. In addition, it was shown dramatically in a mouse study published in 2019 that misfolded α-synuclein in the gut can be delivered to the brain via the vagus nerve to cause PD. A vagotomy completely protected the mice from transmission from the gut to the brain [49]. The United States Vaccine Adverse Reporting System (VAERS) is a national vaccine safety surveillance program maintained by the US government where medical practitioners and patients alike can submit cases of adverse reactions they believe were related to any vaccine they received [50]. An analysis of data from VAERS involved tabulating the counts in the year 2021 of various adverse events that listed symptoms that could be associated with inflammation in the vagus nerve and/or the major nerves in the head that it connects to. These symptoms included anosmia (loss of smell), tinnitus, deafness, facial palsy, vertigo, migraine headache, dysphonia, dysphagia, nausea, vomiting, dyspnea, syncope, and bradycardia. There were over 200,000 cases with these symptoms linked to the COVID-19 vaccines in 2021, representing 97.2% of all the cases for any vaccine linked to these symptoms in that year [17]. While the evidence from VAERS is not proof that there is inflammation in the vagus nerve in association with these symptoms, such inflammation is a compelling plausible explanation for the observed symptoms. There is also evidence that exosomes play an important role in the propagation of amyloidogenic proteins in the brain. The human prion protein, PrP, is found in association with exosomes in both its normal (PrP^C^) and its misfolded (PrP^SC^) form. Furthermore, exosomes containing PrP^SC^ are infectious [51]. Exosomes can transport both amyloid β and phosphorylated Tau, two proteins that are linked to Alzheimer's disease. The Aβ plaques associated with Alzheimer's are enriched in exosomal proteins, suggesting an original source from exosomes [52]. In a mouse model of tauopathy, techniques that inhibit exosome synthesis have been found to arrest tau propagation [53]. Tau protein coaggregates with misfolded Aβ in plaques in the brains of AD patients, and this suggests there may be a general principle of toxicity induced via endocytosis of exosomes [54]. Finally, exosomes specifically derived from cells undergoing Tau aggregation can seed and corrupt soluble Tau in recipient cells [55]. One of the types of molecules often presents in exosomes is microRNAs (miRNAs). miRNAs are small single-stranded non-coding RNA molecules containing around 22 nucleotides that are found across multiple phyla, including animals, plants, and viruses. They play an essential regulatory role through their ability to silence the expression of genes for specific proteins, usually by binding to the 3' and 5' (near the 5' cap) untranslated regions (3',5' UTRs) of the mRNA molecule that codes for the protein [56,57]. Both antigen-presenting dendritic cells (DCs) and T cells can secrete and take up exosomal miRNAs. So it is appropriate to view exosomes as a cell-cell communication mechanism to transfer these essential regulatory RNAs among different cell types in association with other cargo [58]. Two miRNAs that are important for our discussion here are miR-155 and miR-146a. Both have been found present in exosomes released by immune cells upon exposure to endotoxins [58]. Both have also been singled out on the short list of miRNAs whose expression levels are altered in association with COVID-19 [59].

It has been demonstrated experimentally that exosomes play an essential role in cell-cell communication between T cells and B cells during the process of antibody production following antigen presentation in germinal centers. Three specific miRNAs, one of which was miR-155, were identified as being present in these exosomes and were essential for eliciting the appropriate B-cell response. The miRNAs promoted survival, proliferation, and antibody class switching in the B cells, all essential for the antibody production process [60]. We have previously shown how miR-155, in particular, likely plays a role in myocarditis associated with the mRNA vaccines [17]. Here, we will argue for a role for miR-146a in the induction of neurodegenerative diseases. We hypothesize that exosomes released from immune cells in the spleen travel up the vagus nerve to reach the brain stem nuclei. They deliver their toxic cargo, which can include not only the spike protein but also intact mRNA molecules that encode the protein, to recipient cells in the brain. Microglia in the brain, in turn, could take up the spike protein and/or the mRNA, potentially leading to further upregulation of these microRNAs. miR-146a is a commonly expressed miRNA that is involved in many disease states. In particular, it is highly associated with both viral infection and prion diseases in the brain [61,62]. miR-146a has been shown to suppress rho-associated, coiled-coil-containing protein kinase 1 (ROCK1), which results in hyperphosphorylation of Tau in association with Alzheimer's disease [63]. miR-146a suppresses the translation of ROCK1 mRNA into protein by binding to its 3' UTR. It may be confusing that suppressing a kinase leads to increased phosphorylation of Tau, but ROCK1 does not act directly on Tau. ROCK1 phosphorylation of the protein phosphatase and tensin homolog (PTEN) activates PTEN to promote tau dephosphorylation. So, miR-146a suppression of ROCK1 results in PTEN inactivation, which leads to the accumulation of phosphates attached to Tau. Another role for ROCK1 is to repress excessive recruitment of macrophages and neutrophils during acute inflammation, so its suppression by miR-146a results in excessive macrophage and neutrophil infiltration into the tissue, thus increasing inflammation [64].

A review paper by Pogue AI and Lukiw WJ showed that miRNAs are abundant in the brain and participate in the progression of many age-related neurological disorders. miR-146a was singled out as a pro-inflammatory contributory factor in several neurodegenerative diseases, including Alzheimer's disease, amyotrophic lateral sclerosis (ALS), macular degeneration, multiple sclerosis, temporal lobe epilepsy, and CJD [61]. As was mentioned above, miR-146a is upregulated in response to endotoxins. The spike protein contains a sequence just above its furin cleavage site that is a superantigen-like motif sequentially and structurally similar to a segment of enterotoxin B (SEB) produced by *Staphylococcus aureus* [65]. Furthermore, as we will see in the next section, there is a direct signaling pathway through which it can be expected that the spike protein would upregulate miR-146a in microglia receiving the exosomes.

In a previous review publication, we proposed that a major effect of the mRNA vaccines was to inhibit type I interferon (IFN) signaling, leading to increasing susceptibilities to the activation of latent viruses and cancer [17]. This hypothesis is supported by in vitro clinical analysis showing that spike protein interacts with Interferon Regulatory Factor 3 (IRF3) and results in the termination of IFN-I activation [66]. Further in vitro investigations on macaque lung cells obtained by bronchoalveolar lavage reinforces the validity of hypothesizing that spike impairs the type I interferon expression [67]. Based on this in vitro evidence, we suggest that overexpression of miR-146a could be a significant contributory factor in this downregulation. It has been shown that miR-146a suppresses type I IFN signaling through suppression of the synthesis of several signaling proteins, including IRF5, Signal Transducer and Activator of Transcription 1 (STAT1) Interleukin-1 Receptor-Associated Kinase 1 (IRAK-1), and tumor necrosis factor (TNF) receptor-associated factor 6 (TRAF6), all of which are essential mediators of IFN signaling [68].

CD16+ monocytes and toll-like receptor 4

As many as 30% of patients infected with SARS-CoV-2 continue to experience debilitating symptoms long after the virus has cleared. This condition, referred to colloquially as "long COVID," has also been formally named "post-acute sequelae of COVID" (PASC). Common symptoms include breathlessness, fatigue, brain fog, inflammation, and coagulopathies. A study based on 46 individuals suffering from PASC found that two specific non-classical monocyte types, (CD14Lo, CD16+) and (CD14+, CD16+) were significantly elevated in the PASC patients up to 15 months after the acute infection. A statistically significant number of these non-classical monocytes were found to still contain SARS-CoV-2 S1 protein up to 15 months post-infection [16].

A follow-on preprint human study involved individuals who experienced PASC-like symptoms post-vaccination for COVID-19. CD16+ monocytes were isolated from six of these patients, and it was confirmed that they, too, contained both S1 and S2 sequences, as well as several mutant S1 peptides [69]. It was proposed that the continual release of spike protein fragments from these monocytes could sustain the PASC symptoms. It is conceivable that these monocytes have reverse-transcribed the mRNA into DNA, likely stored in plasmids [70]. It has been shown experimentally that human cells expressing the retrotransposon long interspersed nuclear element-1 (LINE-1) are able to reverse-transcribe the spike protein mRNA into DNA within six hours of exposure through transfection [71].

The Gag polyprotein, present in all retroviruses, is an essential nucleic-acid-binding protein that coordinates many aspects of virion assembly as an important step towards reverse transcription and integration into the host DNA [72]. A paper published in 2020 with the provocative title: "Prion protein PrP nucleic acid binding and mobilization implicates retroelements as the replicative component of transmissible spongiform encephalopathy," proposed that PrP is a nucleic-acid-binding antimicrobial protein that, like retroviral Gag proteins, can trigger reverse transcription by binding to LINE-1 retroelement-derived RNA. Furthermore, they claimed that PrP^SC^'s cytotoxicity is dependent upon its ability to facilitate LINE-1 retrotransposition activity [73]. This leads to DNA double-strand breaks and cellular damage, but, as well, it can be inferred that PrP^SC^, and, by analogy, the spike protein itself, which is also an RNA-binding protein, may facilitate the retrotranscription of spike protein mRNA into DNA, mediated by LINE-1. While LINE-1 is inactive in most cells, neurons, like cancer cells and immune cells, actively express LINE-1, especially in association with neurodegenerative diseases [74,75]. The potential implications of all of this are sobering.

Fibrinogen in the blood can clot into an anomalous amyloid form of fibrin that, similar to other β-rich amyloids and prions, is relatively resistant to proteolysis (fibrinolysis). In a human study where blood and platelet-poor plasma samples (PPP) were collected from healthy volunteers and COVID-19 patients, it was concluded that the SARS-CoV-2 spike protein produces blood hypercoagulation and fibrinogen changes in a direct way, even when it is not taken up by cells [76]. A paper by Kell DB et al. provided evidence that the SARS-CoV-2 spike protein can interact with fibrin to form aberrant amyloid fibrin microclots, termed fibrinaloids. These microclots can inhibit the transport of erythrocytes to capillaries, disrupting the supply of oxygen to the affected tissues. They argued that this feature of the spike protein could be the primary underlying etiology of PASC [77]. Indeed, platelets are the largest reservoir of prion protein in the blood. Platelet activation has been shown to induce platelets to release the prion protein and display it on the surface of exosomes [78]. This pathology is strikingly reminiscent of the ability of prion-like proteins to cause misfolding of proteins in the brain leading to neurodegenerative disease, and the underlying biophysical aspects may be analogous. Support for this idea comes from observations of the properties of amyloid β. Amyloid β-associated thromboses have an abnormal structure and are resistant to degradation through proteolysis. Amyloid β interacts with fibrinogen, and its binding to fibrinogen facilitates oligomerization [79]. The authors of this paper argued that these properties of amyloid β might make it an important contributor to the vascular abnormalities that are associated with Alzheimer's disease.

Blood monocytes recognize endotoxins produced by Gram-negative bacteria through a toll-like receptor 4 (TLR4) pathway [80]. The TLR4 pathway induces an inflammatory response by upregulating both mRNA and protein levels for TNF-α and interleukin-1β (Il-1β), mediated via myeloid differentiation factor 88 (MyD88) [81]. The staphylococcal superantigen, enterotoxin B (SEB), is a potent inducer of TNF-α, and it stimulated the great expansion of (CD14Lo, CD16+) monocytes. The addition of recombinant TNF-α to whole blood culture resulted in the expansion of the (CD14Lo, CD16+) monocyte population to 35% of the total monocyte pool [82]. Notably, the SARS-CoV-2 spike protein has a sequence just above the furin cleavage site closely resembling SEB. This sequence is not present in the original SARS-CoV [65].

A carefully conducted experiment has demonstrated convincingly that the SARS-CoV-2 spike protein binds to TLR4 and activates it. The spike trimer directly binds to the TLR4 receptor with an affinity of ∼300nM, comparable to the binding strength of many virus-receptor interactions. Furthermore, the spike protein robustly induces the inflammatory agent Il-1β, and this induction is lost when TLR4 inhibitors are added [83]. It is conceivable that the segment that resembles SEB is responsible for TLR4 activation.

A research paper by a team in Boulder, CO, focused on the S1 subunit of the spike protein and demonstrated that injection of the S1 segment into the cisterna magna of adult male Sprague-Dawley rats resulted in behavioral deficits, microglial activation, and neuroinflammatory response. They determined that S1 signals via a pathogen-associated molecular pattern (PAMP). In vitro experiments on transgenic TLR4 human embryonic kidney (HEK)-293 cells showed that S1 binds to TLR4 receptors to induce upregulation of TNF-α and other proinflammatory cytokines [84].

There is a growing body of evidence supporting the role of TLR4 in PD [85]. TLR4 expression is high in the substantia nigra in association with PD, along with upregulation of the inflammatory cytokine IL-1β [86]. Parkinson's patients also have enhanced expression of TLR4 in circulating monocytes and B cells [87].

**Figure 1:**
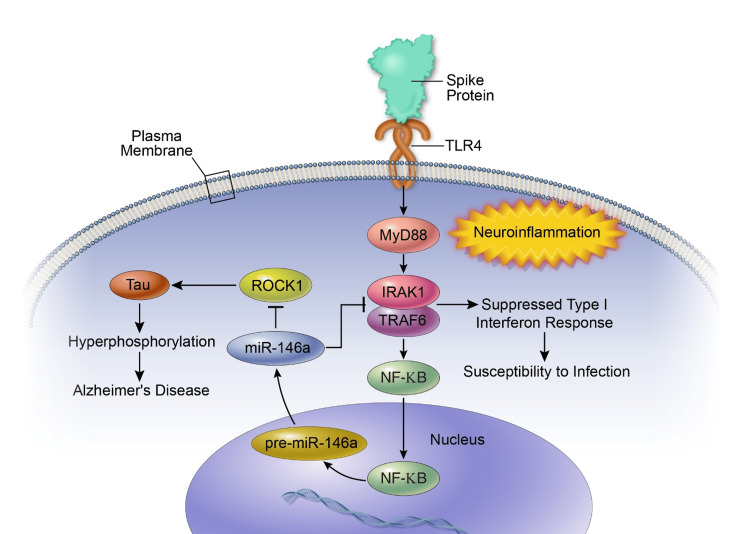
Schematic of pathways and consequences of spike protein binding to the TLR4 receptor in neurons and stimulating the NF-kB signaling response, leading to upregulation of miR-146a and subsequent sequelae. Source: [55,56,58,70,72,73].

Figure 1 schematizes the proposed pathways involved in spike protein activation of the TLR4 signaling response and upregulation of miR-146a in neurons.

Severe COVID symptoms share many features with sepsis [88]. The CD16+ monocyte subset is expanded in sepsis patients, and a dysregulated inflammatory response in CD16+ monocytes is linked to sepsis [80]. Sepsis patients have elevated levels of CD16+ monocytes in their blood, which is associated with elevations in the inflammatory chemokine Il-6 [89].

The (CD14+, CD16+) monocytes have been recognized as exhibiting higher expression of proinflammatory cytokines and higher potency in antigen presentation than other monocytes. As such, they play a crucial role in infection and inflammation [90]. In a study on patients suffering from AIDS dementia, it was found that (CD14+, CD16+) cells represented an extremely high percentage (37% on average) of the monocytes in their blood, compared with only 6.5% in HIV-negative controls. These authors indicated that these cells "might enter the brain and expose neural cells to toxic factors." [91]. A relevant research study by Prabhu VM et al., 2019, has provided solidly convincing results indicating that the monocyte subtype levels (i.e., monocyte dysregulation) correspond with what is observed in long-term non-progressor (LTNP) HIV activation [92]. Although the CD4 counts in these patients are within the normal range, they are prone to a lengthened systemic inflammation and a high non-T-cell correlated viremia risk. More precisely, the non-classical CD14+, CD16+ expansion of monocytes in this study was tightly correlated with what is observed in LTNP HIV-positive patients. In these patients, very high expression of the mannose receptor CD206 characteristic of an M2 phenotype on monocytes, together with increased T-cell activation, corresponded to an increase in the viral load. Thus, the non-classical monocyte subtype in LTNP HIV patients is characterized by a chronic T-cell inflammatory response.

Furthermore, abnormally high levels of (CD14+, CD16+) monocytes are also associated with sarcoidosis and complex regional pain syndrome, a condition associated with neurogenic inflammation [93,94]. Sarcoidosis is a disease of chronic inflammation characterized by granuloma formation. In sarcoidosis, the monocytes and cells of monocyte origin are increased in numbers compared to healthy controls. Moreover, the increased TNF expression in sarcoidosis patients is due to the monocytes present, as they are found in high numbers in numerous bronchioalveolar lavage (BAL) samples of multiple patients. Therefore, the clinical severity of sarcoidosis patients is tightly linked to the number of monocytes present [95]. However, another research study by Fraser SD et al., 2020, concludes that the non-classical monocyte type in sarcoidosis is expanded and that they are characterized by very low expression of regulatory receptors [96].

We recognize that the connections we are proposing between these cellular events and the clinical manifestation of inflammation and specific pathologies rely upon an inferential chain that links in vitro and in vivo data to histological findings in human subjects. Nevertheless, we believe it is important to consider these potential connections so they might be more rigorously investigated.

Potential interactions between the spike protein and PrP

The AIDS virus, HIV, invades the central nervous system where it causes neuroinflammation leading to cognitive impairments. A study published in 2017 demonstrated that TNF-α expression, induced by HIV, led to the shedding of PrP from astrocytes in the brain. The levels of PrP in the cerebrospinal fluid of AIDS patients suffering from cognitive issues were elevated compared to those of AIDS patients without cognitive issues [97].

The S2 segment of the spike protein is responsible for membrane fusion of viral and cellular membranes. A study of the 3D structural aspects of S2 and the gp41 protein from HIV-1 revealed that these two proteins share the same two α helices and could follow an analogous membrane fusion mechanism [98].

The fact that the spike protein induces sharp TNF-α upregulation and causes cognitive issues implies that it might also, like HIV, upregulate PrP expression in the brain. Recently, our team has shown that the expression of PrP^C^ is induced due to spike protein interactions in murine microglia, and human monocytes and macrophages [38]. In this study, it is predicted that, according to the toll-like receptor activation by the spike protein, both the normal isoform of the prion protein and the expression of β amyloid are upregulated through the promotion of p53 expression via β amyloid metabolism. An increase in PrP^C^ numbers can lead to prion conformation misfolding and generates prion and prion-related diseases [99,100].

While it is unclear what the primary function of the prion protein is, it has been shown that it is protective under neuronal stress conditions. PrP expression is increased in the plasma of stroke patients, and it protects neurons from apoptosis [101]. Also, there is evidence that PrP protects cells under oxidative stress conditions from senescence. Induction of senescence in fibroblasts grown in culture through incubation with copper sulfate resulted in an increase in PrP mRNA levels, an increase in PrP protein abundance, and a nuclear localization of PrP. Knockdown of PrP expression through small interfering RNA resulted in an increase in markers of senescence. The conclusion from these findings is that PrP is upregulated under oxidative stress conditions and it helps as an antioxidant to delay senescence transformation [102].

The spike protein has been shown experimentally to induce senescence in transfected cells [103]. Furthermore, it has been proposed that the mRNA vaccines can induce premature senescence via syncytia formation in exposed immune cells, due primarily to their lipid content [104]. The vaccines contain ionizable lipids, cholesterol and the phospholipid 1,2-distearoyl-sn-glycero-3-phosphocholine (DSPC), all of which can contribute to syncytia formation. DSPC is a mimetic of phosphatidyl serine (PS), and it is well established that externalized PS promotes syncytia formation [105]. A mouse model has demonstrated that senescent immune cells drive systemic aging in mice, and likely also in humans [106].

*In vitro* molecular study shows that macromolecular crowding can facilitate the conversion of native PrP into the neurotoxic soluble β oligomer configuration [107]. It is conceivable that the rapid production of spike protein from the mRNA in transfected immune cells would induce a crowded environment, while at the same time upregulating PrP synthesis due to the stressful condition. This could be an ideal environment for the formation of PrP^SC^ molecules, which would be released within exosomes from transfected immune cells in the spleen and elsewhere. The transformation of PrP^C^ to the infectious PrP^SC^ molecule is an extremely slow process in the absence of PrP^SC^. However, under the influence of intermediate PrPs, PrP^SC^ can induce solid and irreversible amyloid genesis via the template-assistance model. In another model, where PrP^SC^ is present and interacts with PrP^C^, the progression to amyloid genesis is fast and reversible (nuclear-polymerization model), to establish neurotoxicity [99,100].

**Figure 2:**
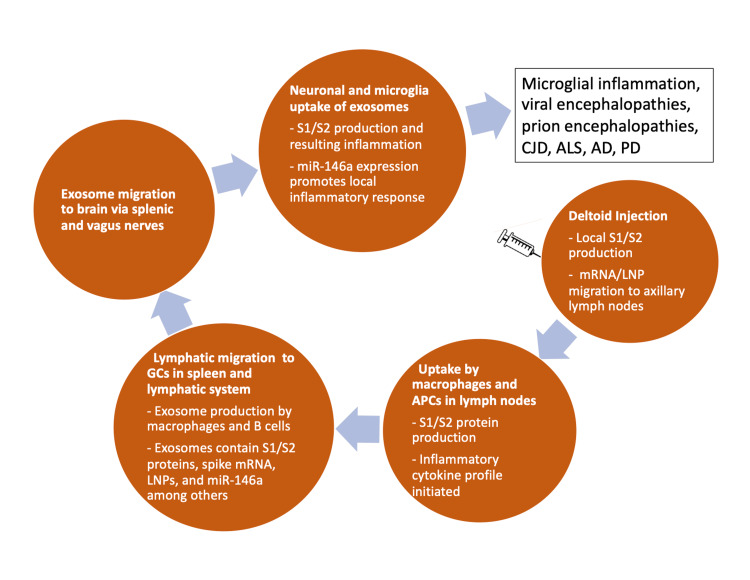
Schematic of the sequelae to mRNA injection into the deltoid muscle, ultimately leading to neurodegeneration in the brain. APCs: Antigen-presenting cells; LNPs: Lipid nanoparticles; CJD: Creutzfeldt-Jakob disease; ALS: Amyotrophic lateral sclerosis; AD: Alzheimer’s disease; PD: Parkinson’s disease.

Figure 2 shows a schematic of the likely sequence of events leading to neurodegeneration, beginning with the injection in the deltoid muscle.

A role of Hsp70 and diabetes

Multiple studies in humans have shown that people who suffer from diabetes and/or obesity have an increased risk of severe outcomes from COVID-19 [108,109]. One possible explanation for this observation is that these conditions disrupt the heat shock response (HSR), a natural response to a fever that normally leads to the resolution of the inflammatory response [110-112]. In fact, high-risk COVID-19 patients have a suppressed anti-inflammatory HSR [113]. Heat shock transfer factor 1 (HSF1) is the major transcription factor regulating the expression of heat shock proteins. It suppresses the activity of Il-6 and Il-1β, thus taming the inflammatory response [114]. It is often excessive cytokine production by an overactive immune system that leads to tissue damage and life-threatening multiple organ failure [115].

Normally, HSR induces the expression of inducible heat shock protein 70 (Hsp70), also known as Hsp72 and Hspa1a, a molecular chaperone with many complex roles in metabolism and regulatory processes. Heat shock proteins can account for up to 2% of the total protein mass in a cell following activation by HSR [116]. Hsp70/72 interacts with many other proteins during the protein folding process to facilitate folding, helping to protect from the formation of protein aggregates and facilitating the degradation of damaged proteins [117].

Stressful stimuli can induce the release of intracellular heat shock proteins into the extracellular milieu and circulation. Extracellular Hsp70/72 plays a facilitative role in the adaptive immune response to antigens [118]. Also, extracellular Hsp70/72 can bind antigens, and the complex is then recognized by antigen-presenting cells (APCs) via scavenger receptors. The APC takes up the complex, and bound Hsp70/72 sequesters the antigen until it reaches the proteasome. After processing, the antigen is transported to MHC class I molecules, triggering the activation of cytotoxic CD8+ T cells. The Hsp70/72-antigen complex can also be processed in the lysosome, leading to the presentation of antigen-derived peptides on MHC class II molecules, thus activating CD4+ T cells [119,120].

Impaired insulin signaling leads to a deficient ability to induce HSR and the subsequent resolution of inflammation. Glycogen synthase kinase-3 (GSK-3) is a serine/threonine kinase that plays essential role in the molecular pathophysiology of many diseases. Its overexpression is linked to insulin resistance [121]. GSK-3 negatively regulates both the DNA-binding and transcriptional activities of HSF1 [122]. The promoter region of the TNF-α gene contains a binding site for HSF1 that represses TNF-α transcription. Therefore, those with insulin resistance face an increased susceptibility to endotoxin exposure due to their impaired ability to induce HSF1 expression [123].

One of the most important functions of Hsp70 is to protect from neurodegenerative disease. Many papers in the research literature link Hsp70 to protection from various protein-misfolding neurological diseases through its ability to facilitate proper folding and delay fibril formation [124-126]. Evidence from in vitro studies is also very clear. Pharmacological induction of Hsp70 in cells chronically infected with prions significantly decreased PrP^SC^ accumulation. Furthermore, mice lacking the gene for Hsp70 experienced accelerated prion disease progression compared with wild-type mice [126].

Human post-mortem studies from patients who died from COVID-19 have shown that a primary target of the SARS-CoV-2 spike protein is immune cells, particularly macrophages, in the spleen and that many infected cells committed apoptosis [127]. Apoptotic cells release higher numbers of exosomes than healthy cells [128]. Although a thorough in vitro and in vivo investigation is necessary to confirm this, we can predict that immune cells in the germinal centers in the spleen constantly synthesizing spike protein under the instruction of the mRNA in the mRNA vaccine would be under considerable stress due to the excess protein load and the potential for spike protein fragments to misfold into an amyloidogenic form. Pyrexia (fever) is a very common adverse reaction to the vaccine, indicating activation of the HSR. The immune cells in the spleen would be expected to upregulate Hsp70 under the influence of HSF1. They would likely release it into exosomes, along with the spike proteins and the miRNAs, such as miR-155 and miR-146a, needed to trigger an appropriate antibody response to spike.

Exosomes represent a novel and efficient method for prion transmission. Stimulation of exosome release increases the intercellular transfer of prion proteins, and conversely, pharmacological inhibition of exosome release decreases prion transfer efficiency [129]. Vaccinated obese or diabetic people would suffer from an impaired ability to instantiate the HSR, leaving cells taking up exosomes containing the spike protein less protected from spike protein misfolding and, therefore, more vulnerable to apoptosis, creating a vicious cycle.

A potential role of G quadruplexes

A consideration in comparing the vaccine spike protein to the protein synthesized by the virus is related to the "codon optimization" step in specifying the mRNA for the vaccines. This practice takes advantage of the redundant nucleotide codes for most amino acids, and it involves replacing the codons used by the virus with ones that are more efficient in protein assembly. It turns out that the most efficient codons, on average, contain more guanines than other codons. Guanine nucleotides, when they are enriched in the nucleotide sequence, are sometimes able to configure into a special structure called a "G quadruplex" (G4) [17]. G4s have become a hot topic recently due to their potential ability to regulate translation in poorly understood ways [130]. In addition, it has become apparent that the human prion protein's mRNA contains multiple G4-forming motifs. It has been hypothesized that G4s may play a critical role in causing the prion protein to assume its misfolded state [131]. The original nucleotide sequence in the virus version of spike protein mRNA only has the potential to form four G4 motifs. In contrast, the Pfizer version has the potential to produce nine, and the Moderna version can form 19 [132]. The author of a paper published in 2014, aptly titled "G-quadruplexes within prion mRNA: the missing link in prion disease?" wrote the following in the conclusion: "The presence of G4 forming motifs in PrP mRNA may provide the missing link in the initial conversion of PrP^C^ to PrP^SC^. Understanding how mRNA structures are involved in the (mis-)folding of PrP^C^ and possibly many other RNA-binding proteins with prion-like properties is of prime importance for the development of better treatments of CJD and related diseases" [131].

Reassessment of the risk/benefit ratio of COVID-19 vaccination

A study published in the Lancet tracked the effectiveness of COVID-19 vaccines over time. It showed that once eight months had elapsed since the second injection of the two-injection series, immune function was lower than that among unvaccinated individuals [133]. While boosters can temporarily restore higher levels of antibodies, frequent boosters could further erode innate immune function for an indefinite period, leading to an increased risk of various infections and cancer. Furthermore, the rapid evolution of the virus is resulting in ever-weakening antibody binding to the spike protein of the now dominant strain. Fortunately, the current Omicron strain of the virus appears to be less virulent than the original one. This may be a consequence of the decreased potential for prion-like misfolding.

We acknowledge that we have relied heavily on in vitro and in vivo studies. However, we link these as closely as possible with the documented human histological and pathological correlates with cellular pathways and events. In light of these considerations, the risk/benefit ratio for the mRNA vaccines needs to be reevaluated. With every vaccine comes a flood of spike protein released into the circulation, further advancing the potential for amyloidogenic effects and increasing the risk of future neurodegenerative disease. A comment by Kenji Yamamoto published in BMC is urging the medical community to keep track of the date of the most recent vaccination of hospital patients in order to be better able to assess what role the vaccine may have played in any manifest disease or condition. He also strongly discourages policy that promotes continued boosting of anyone other than the most at-risk patients to death from COVID-19 [134]. There is an urgent need for governments to reconsider a blind policy that assumes that repeated vaccine boosters are a valid approach to dealing with COVID-19.

## Conclusions

We have examined the extensive literature concerning the prion-like properties of the SARS-CoV-2 spike glycoprotein. Furthermore, we identify pathways through which the mRNA vaccines could be capable of delivering the spike protein to the brain, which we suggest happens via exosomes released from germinal centers in the spleen traveling up the vagus nerve, increasing the risk of neurodegenerative disease. Should this happen, it would be expected that the COVID-19 vaccines would shorten the time period before neurodegenerative disease manifests in susceptible individuals. We speculate that the age of onset of neurodegenerative disease at the population level will decrease in the future in countries where vaccine uptake has been high.

Particularly concerning is the evidence that CD16+ monocytes can continuously produce spike protein for months after vaccination, possibly through prolonged cytosolic presence of mRNA or reverse transcription of the mRNA into DNA. It has become clear that the antibodies induced through vaccination wane over time, necessitating frequent boosters to raise the antibody levels for sufficient protection from COVID-19. With each booster comes a compounded risk of future neurodegenerative disease. Fortunately, Omicron variant infection has a greatly reduced prion-like capability, and thus, with the cessation of mass vaccination, the expected increase in prion-like diseases could stabilize over the coming years.
